# Targeting M-MDSCs enhances the therapeutic effect of BNCT in the 4-NQO-induced murine head and neck squamous cell carcinoma model

**DOI:** 10.3389/fonc.2023.1263873

**Published:** 2023-10-02

**Authors:** Chun-Hsiang Chang, Chi-Jui Chen, Ching-Fang Yu, Hui-Yu Tsai, Fang-Hsin Chen, Chi-Shiun Chiang

**Affiliations:** ^1^ Department of Biomedical Engineering and Environment Sciences, National Tsing Hua University, Hsinchu, Taiwan; ^2^ Institute for Radiological Research, Chang Gung University, Taoyuan, Taiwan; ^3^ Department of Radiation Oncology, Chang Gung Memorial Hospital Linkou Branch, Taoyuan, Taiwan; ^4^ Institute of Nuclear Engineering and Science, National Tsing Hua University, Hsinchu, Taiwan

**Keywords:** BNCT, MDSCs, 4-NQO, CSF-1R inhibitor, HNSCC

## Abstract

**Purpose:**

Malignant head and neck squamous cell carcinoma (HNSCC) is characterized by a poor prognosis and resistance to conventional radiotherapy. Infiltrating myeloid-derived suppressive cells (MDSCs) is prominent in HNSCC and is linked to immune suppression and tumor aggressiveness. This study aimed to investigate the impact of boron neutron capture therapy (BNCT) on the MDSCs in the tumor microenvironment and peripheral blood and to explore the potential for MDSCs depletion combined with BNCT to reactivate antitumor immunity.

**Methods and materials:**

Carcinogen, 4-NQO, -induced oral tumors were irradiated with a total physical dose of 2 Gy BNCT in Tsing Hua Open Reactor (THOR). Flow cytometry and immunohistochemistry accessed the dynamics of peripheral MDSCs and infiltrated MDSCs within the tumor microenvironment. Mice were injected with an inhibitor of CSF-1 receptor (CSF-1R), PLX3397, to determine whether modulating M-MDSCs could affect mice survival after BNCT.

**Results:**

Peripheral CD11b^+^Ly6C^high^Ly6G^-^ monocytic-MDSCs (M-MDSCs), but not CD11b^+^Ly6C^lo^Ly6G^high^ polymorphonuclear-MDSCs (PMN-MDSCs), increased as tumor progression. After BNCT treatment, there were temporarily decreased and persistent increases of M-MDSCs thereafter, either in peripheral blood or in tumors. The administration of PLX-3397 hindered BNCT-caused M-MDSCs infiltration, prolonged mice survival, and activated tumor immunity by decreasing tumor-associated macrophages (TAMs) and increasing CD8^+^ T cells.

**Conclusion:**

M-MDSCs were recruited into 4-NQO-induced tumors after BNCT, and their number was also increased in peripheral blood. Assessment of M-MDSCs levels in peripheral blood could be an index to determine the optimal intervention window. Their temporal alteration suggests an association with tumor recurrence after BNCT, making M-MDSCs a potential intervention target. Our preliminary results showed that PLX-3397 had strong M-MDSCs, TAMs, and TIL (tumor-infiltrating lymphocyte) modulating effects that could synergize tumor control when combined with BNCT.

## Introduction

Head and neck (H&N) cancers are the sixth leading cancer worldwide (1). This cancer comprised heterogeneous groups of tumors arising from the mucosal surfaces of the upper aerodigestive tract, including sinonasal, oral cavities, nasopharynx, oropharynx, hypopharynx, and larynx (2). Among H&N cancers, 90% are head and neck squamous carcinoma (HNSCC). The mainstay of treatment for HNSCC is surgery, adjuvant radiotherapy, concurrent chemoradiation, new immunotherapy, or combination therapies (3–7). Despite advances in medicine, the 5-year survival rate has only remained at approximately 50% for HNSCC patients (8). Local, regional recurrence is noted in 14% of patients with HNSCC (9), the primary cause of patient death. Although advanced techniques in radiotherapy, such as intensity-modulated radiotherapy (IMRT) and stereotactic radiotherapy, could improve tumor control rates in local recurrent patients (10, 11). However, the prognosis of those recurrent patients is still unsatisfactory (10, 12, 13).

Boron neutron capture therapy (BNCT) is a binary treatment that combines the selective accumulation of boron carriers in tumors and irradiation with epithermal/thermal neutron beams (14). After bombarding with low energy (<0.5 eV) neutrons, ^10^B absorbs the neutron, and further disintegrates into alpha (^4^He) particles and a recoiled lithium nucleus (^7^Li) (15), both depositing its high energy along their short path (<10 μm) (16). The path lengths of these particles are approximately one cell diameter, making BNCT a potentially ideal way to selectively destroy malignant cells and spare normal tissues if a sufficient number of ^10^B is uptaken by tumor cells (17). Up to now, BNCT has been clinically applied for treating glioblastoma multiforme, melanoma, liver metastasis, and head and neck cancers among others in several countries (18–20). Data from clinical trials in Taiwan revealed that BNCT results in a high response rate for locally recurrent head and neck and malignant brain tumor patients (21, 22). BNCT exerts a profound effect on tumor control, but the impact on the tumor microenvironment (TME) and the potential to combine with immunotherapy is rarely discussed.

Myeloid-derived suppressor cells (MDSCs) are a heterogeneous population of myeloid cells that comprise a significant component in TME and can modulate immune response (23, 24). MDSCs consist of three major subpopulations, termed polymorphonuclear MDSCSs (PMN-MDSCs), monocytic MDSCs (M-MDSCs), and early-stage MDSCs (e-MDSCs) (25). Both PMN-MDSCs and M-MDSCs subsets present specific markers for identification, while e-MDSCs need further identification (26). It has shown that a significant accumulation of MDSCs in the peripheral blood of HNSCC patients (27) and the number of MDSCs infiltrates in tumors was associated with poor prognosis (28). Chemoradiotherapy considerably altered the composition and function of immune cells in that increased level of circulating MDSCs, CD8^+^ effector T cells, and Treg was found in HNSCC patients (29, 30). MDSCs express CSF-1 receptors (CSF-1R; CD115) are actively polarized and recruited to the tumor microenvironment (31, 32). The administration of CSF1/CSF-1R inhibitors to blockage MDSCs was an effective monotherapy for tumor control and adjuvant therapy to overcome treatment resistance (33). Pexidartinib, an orally administered tyrosine kinase inhibitor with potent and selective activity against CSF-1R and the capability of modulating tumor microenvironment (34, 35), has been approved by FDA for patients with giant cell tumors (36).

This study used 4-NQO (4-Nitroquinolone-1-oxide) to induce an oral cancer model on C57BL/6J mice. BNCT treatment had a profound therapeutic effect on tumor control and significantly prolonged mice survival, but it also led to a rise of M-MDSCs in both circulation and tumors. We hypothesized that targeting MDSCs by inhibiting the CSF-1R axis could hinder the M-MSDCs recruitment and activate the immunity in the tumor microenvironment when combined with BNCT, leading to a synergistic effect for better tumor control.

## Materials and methods

### Orthotopic tongue tumor model

Male C57BL/6 mice were obtained from the National Laboratory Animal Center, Taiwan, and used at the age of 8-10 weeks. All animal experiments were performed according to the guidelines and approved by the Institutional Animal Care and Use Committee of National Tsing Hua University (NTHU-110050). A tongue tumor was induced by 4-Nitroquinoline 1-oxide (37, 38) (4-NQO, N0250, TCI, Kita city, Tokyo, Japan) in drinking water (100 μg/mL) for 16 weeks (39), followed by normal drinking water thereafter. The 4-NQO carcinogen induced a slow and multistage carcinogenicity, and the pathological stages underwent hyperplasia, dysplasia, papilloma to invasive squamous cell carcinoma, which led to several lesions on the tongue.

### BNCT dosage simulation

All simulated materials followed the ICRU 46 report. The total absorbed dose rate (Gy/minute) was calculated with a Monte Carlo N-Particle (MCNP) Transport Code. A relative biological effectiveness (RBE) factor of 3.2 was used for Tsing Hua Open Reactor (THOR) epithermal neutron beam. The compound biological effectiveness (CBE) factor of 3.8 was used (40, 41) for tumor tissue. In BNCT, the total weighted dose (Gy(w)) was derived from equation:


$$D_{w}=w_{B}\times D_{B}+W_{n}\times D_{n}+W_{r}\times D_{r}$$

Where D_B_, D_n_, and D_r_ are boron dose, neutron dose, and gamma-ray dose, respectively. The W_B_ is the CBE value of the boron dose, and W_n_ and W_r_ are the RBE values of the neutron and gamma-ray dose, respectively. The total tumor dose was estimated to a total physical dose of 2 Gy.

### BNCT irradiation

The tumor-bearing mice were intraperitoneally injected with 350 mg/kg L-BPA (L*-4-*Boronophenylalanine, GHP-001, TBI, Taoyuan, Taiwan) one hour before irradiation. The time selection was based on our preliminary biodistribution data showing that tumor has the highest uptake at one hour with the ratio of 1.62 to dose limiting tissue (mucosa) (**Supplementary 1**). It is worth reminding that the mucosa in the 4NQO-induced tumor model was also exposed to the carcinogen. It was a precancer tissue (42) and could be more radiosensitive than the mucosa in normal mice (43). The radiation may damage the oral mucosa, leading to oral mucositis (44), a frequent side effect for advanced head and neck cancers (45). Therefore, the mucosa in tumor-bearing mice was chosen as the dose limiting tissue (46). Mice were anesthetized using a mixture of Zoletil (20 mg/mL) and xylazine (Rompun, 8 mg/mL) and restrained on an HDPE (high-density polyethylene) plastic holder. The oral cavity was irradiated with a total physical dose of 2 Gy by 1.2-MW epithermal neutron beam with a flux > 1.3 ×10^9^ n/cm^2^/s from THOR, Hsinchu, Taiwan, and the neutron flux was monitored by NeuTHOR on-line system during the irradiation to achieve a total physical dose of 2 Gy (47).

### Flow cytometry analysis of peripheral blood

The blood sample was withdrawn from submandibular blood without anesthesia and then treated with red blood cell lysis buffer (00-4300-54, eBioscience) for 5 minutes at room temperature. The blood cells were blocked with anti-mouse CD16/32 (Fc block) antibody (553142, BD Biosciences) and then labeled with specific antibodies: anti-mouse CD115 (CSF-1R)-BV605 (743640, BD Biosciences), CD45 -PE-Cy™7 (552848, BD Biosciences), CD11b-PerCp Cy5.5 (561114, BD Biosciences), Ly6G-PE (551461, BD Biosciences), and Ly6C-FITC (553104, BD Biosciences). CD45 was employed to categorize leukocytes. CD11b was utilized to distinguish myeloid-derived cells. Ly6C and Ly6G were used to classify the PMN-MDSCs (Ly6C+ Ly6G+) and M-MDSCs (Ly6C+Ly6G-). The stained cells were analyzed by FACSFortessa flow cytometry. Data was acquired by BD FACSDive™ software and analyzed by FACSDiva™ and FlowJo version 10.6.2.

### Immunofluorescence staining

Tumors were frozen in optimal cutting temperature compound (Tissue-Tek, SAKURA) before cryosectioning. Tumor sections were stained with specific primary antibodies, including rat anti-mouse Ly6C (128001, BioLegend), rabbit anti-mouse Ly6G (87048, Cell Signaling), rat anti-mouse CD68 (MCA1957CA, Bio-Rad), rat anti-mouse F4/80 (MCA497GA, Bio-Rad), rabbit anti-mouse CD8 (ab217344, Abcam), and rabbit anti-mouse FOXP3 (ab54501, Abcam). Primary antibodies were detected by goat anti-rat IgG conjugated with Alex Fluor 488 (A11006, Invitrogen) or by goat anti-rat IgG conjugated with Alex Fluor 594 (A11012, Invitrogen). The images were analyzed by Image-Pro plus 6.0 software (MediaCyberneticsm Rockville, MD, USA).

### Administration of CSF-1R inhibitor

Pexidartinib (PLX-3397) (HY-16749, MedChemExpress) was prepared in distilled water containing 5.6% DMSO (Sigma-Aldrich) and 27.8% PEG-300 (Polyethylene glycol 300, 1546423, Sigma-Aldrich). One week after BNCT, the tumor-bearing mice were treated with 50mg/kg Pexidartinib by intragastric injection once per two days for two weeks.

### Statistical analysis

All statistical analyses and graphs were generated with GraphPad Prism version 8.3.0 (GraphPad Software). Two-tailed Spearmen’s statistics were used for the correlated histoscore. Simple linear regression was used for goodness of fit and calculated the R squared. A curve comparison of the Mantel-Cox test was used for the survival curve. The T-test was used to determine the statistical significance. *P* values less than 0.05 were considered statistically significant (*P< 0.05, **P< 0.01, ***P< 0.001, ****P< 0.0001). All the results are expressed as the mean ± SD with a significant set at p<0.05.

## Results

### Circulating M-MDSCs were correlated to tumor progression in 4-NQO-induced HNSCC mice

**Figure 1:**
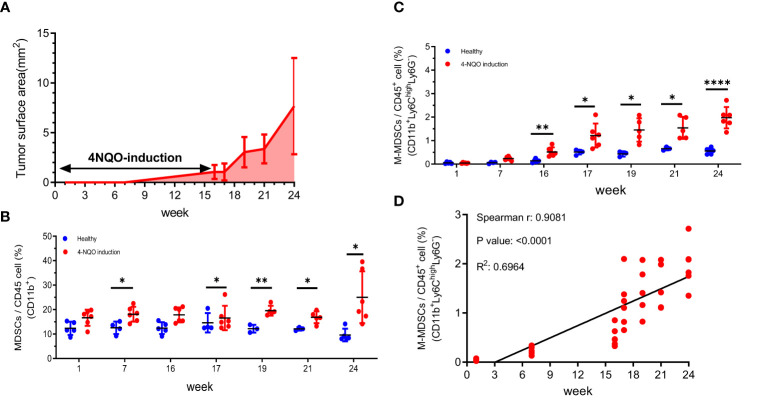
The tumor progression and the changes of circulating MDSCs in a 4-NQO-induced murine tumor model **(A)** The quantification of the progression of oral lesions in 4-NQO-induced mice (N=3 to 5 animals for every time point) The percentage of **(B)** MDSCs (CD11b^+^) and **(C)** M-MDSCs (CD11b^+^ Ly6C^high^Ly6G^-^) among the circulating CD45^+^ population were quantified by flow cytometry during the tumor progression Blue indicates the age- matched healthy mice, and red indicates the 4-NQO group. All quantitative data represented mean ± SD (Unpaired Student's t-test, *P < 0.05, **P < 0.01, ****P < 0.0001). **(D)** The correlation of M-MDSCs with tumor progression at weeks 1, 7, 16, 17, 19, 21, and 24.

4-NQO is an effective carcinogen to induce tongue cancer in C57/B6 mice, displaying similar pathology and morphology to human oral cancer (48). After induction, the tumor progression was examined by surface tumor area once per week. Tumors were barely detected before 7 weeks, slowly progressed between 7 to 16 weeks, and exponentially developed during the withdrawal of 4-NQO from 16 to 24 weeks (**Figure 1A** **)**. Along with the tumor progression, MDSCs subsets in the peripheral blood were examined at indicated time points. The CD11b^+^ MDSCs were measured during the tumor progression by flow cytometry. Flow cytometry verified a significant increase of MDSCs after 7 weeks of treatment with 4-NQO and a continuous expansion even after 4-NQO withdrawal (**Figure 1B** **).** The MDSCs population was further categorized into two main subpopulations: CD11b^+^Ly6C^high^Ly6G^-^ M-MDSCs and CD11b^+^Ly6C^lo^Ly6G^high^ PMN-MDSCs (49). The findings indicated a significant increase in M-MDSCs over a 16-week 4-NQO induction period (**Figure 1C**). The number of M-MDSCs population has a good correlation with tumor progression (**Figure 1D**). In contrast, the number of PMN-MDSCs was no significant alterations and not correlated with tumor progression (**Supplementary 2**). These results suggested that circulating M-MDSCs might be a suitable index for monitoring tumor progression.

### BNCT prolonged the mice’s survival and led to sequential changes of M-MDSCs in peripheral blood and tumors

**Figure 2:**
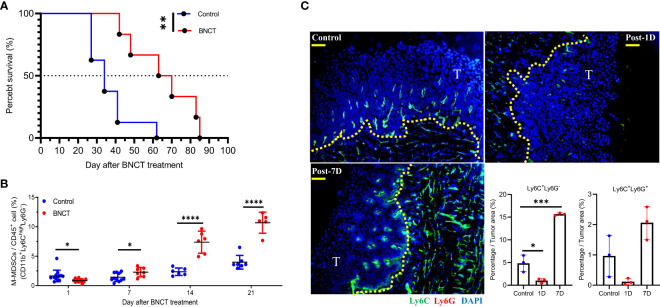
The response of 4 NQO-induced HNSCC to BNCT. **(A)** Survival of tumor-bearing mice left untreated (medium survival=34 ± 12, n = 8) or receiving BNCT treatment (medium survival = 67 ± 18, n = 6). **(B)** The variability of M-MSDCs in the blood with time after BNCT treatment. **(C)** The representative images of control, 1-day post-BNCT (Post-1D), and 7-day post-BNCT (Post-7D) tumors were stained with Ly6C(Green), Ly6G(Red), and nucleus (Blue) (Bar=50 μm), T = tumor, and the quantified percentage of Ly6C^+^Ly6G^-^ and Ly6C^+^ Ly6G^+^ cells within tumors. All quantitative data represented mean ± SD. (Unpaired *t-test*, *P < 0.05, **P < 0.01. ***P < 0.001, and ****P < 0.0001).

BNCT was given to tumor-bearing mice 24 weeks after tumor induction to test its therapeutic efficacy. The BNCT remarkably reduced the tumor area, resulting in a 1.4-fold decrease compared to conventional electron accelerator treatment in an initial study using 6 Gy of absorbed dose (**Supplementary 3**). Unfortunately, the tumor-bearing mice experienced severe side effects that hindered their eating ability, making it challenging to observe their medium survival. Therefore, we took a small dosage of BNCT in followed approach. A total physical dose of 2 Gy BNCT irradiation significantly prolonged mice median survival to 67 ± 18 days in comparison to 34 ± 12 days in control group (p<**, log-rank test) (**Figure 2A** **)**.

After irradiation, there was a mild but significant decrease of peripheral M-MDSCs at one day, compared to the control group. A sequential increase of M-MDSCs was found later at 7 and 14 days (**Figure 2B** **)**. For PMN-MDSCs, there were no significant alterations after BNCT treatment (data not shown). The tongue tumor tissues were also collected and examined for M-MDSCs and PMN-MDSCs subsets by staining with Ly6C and Ly6G markers, respectively. The Ly6C^+^Ly6G^-^ M-MDSCs were eliminated in BNCT-treated tumors immediately at one day and rebounded at 7 days compared to control tumors. Although the changes of Ly6C^+^ Ly6G^+^ PMN-MDSCs were similar to those of Ly6C^+^ Ly6G^-^ M-MDSCs after BNCT treatment, their abundance within the tumor was much lower than M-MDSCs. M-MDSCs were the dominant population within this carcinogen-induced tongue tumor (**Figure 2C**). These results suggested that BNCT triggered a systemic and consistent alteration of the M-MDSCs subset in both peripheral blood and tumor microenvironment. Above findings indicate that peripheral M-MDSCs could be a potential index to monitor the HNSCC tumor progression and the dynamic M-MDSCs infiltrates within the tumor microenvironment before and after BNCT treatment.

### BNCT synergized with CSF-1R inhibitor to improve tumor control and caused a decrease in M-MDSCs numbers in the blood

**Figure 3:**
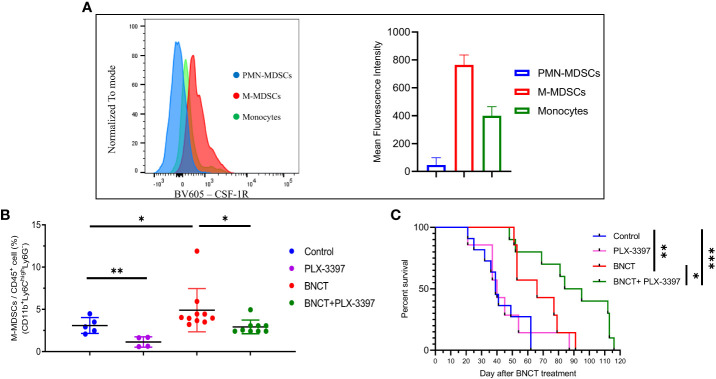
The effect of PLX-3397 (CSF-1R inhibitor) on tumor progression and the frequency of M-MDSCs. **(A)** The mean fluorescence intensity of CSF-IR in the subtypes of myeloid-derived cells. **(B)** The variability of circulating M-MSDCs after treatments. (mean ± SD, unpaired *t* test, *P < 0.05, **P < 0.01). **(C)** Survival of tumor-bearing mice left un-treated (medium survival = 39 ± 14 days, n = 11), receiving PLX-3397 treatment (medium survival = 40 ± 21 days, n = 7), BNCT treatment (medium survival = 66 ± 16 days, n = 7), and combined therapy (medium survival = 90 ± 26 days, n = 10). (logrank test, *P < 0.05, **P < 0.01, ***P < 0.001).

Due to the immunosuppressive role of general MDSCs and their rapid rebound in one week after BNCT, strategies against MDSCs trafficking or functionality were proposed to combine with BNCT treatment to improve tumor control. Colony-stimulating factor 1 receptor (CSF-1R) was suggested to be the potential target for intervention because of its general high expression on the surface of myeloid cells to mediate their trafficking and differentiation (50). Peripheral blood was collected for examination of the CSF-1R expression on each subset of myeloid cells, in which Ly6C^high^Ly6G^-^ M-MDSCs exhibited the highest expression level while the Ly6C^-^Ly6G^-^ monocytes had only half and Ly6C^lo^Ly6G^high^ PMN-MDSCs had the lowest, approximately 5%, compared to Ly6C^high^Ly6G^-^ M-MDSCs. (**Figure 3A**). A CSF-1R inhibitor, namely PLX-3397, was tested on tumor-bearing mice and examined for its efficacy in tumor control. Mice were left untreated or treated at week 19, after 4-NQO induction for solid tumor establishment, using three regimens: (i) BNCT was applied as monotherapy, (ii) PLX-3397 was administered by oral gavage once per two days and lasted from day 7 to day 21 or (iii) mice were subjected to combination therapy, in that PLAX-3397 was administered one week after BNCT. The analysis of blood samples revealed that the administration of PLX-3397 could effectively decrease M-MDSCs level in control mice and BNCT-induced M-MDSCs level at 21 days (**Figure 3B**).

**Figure 4:**
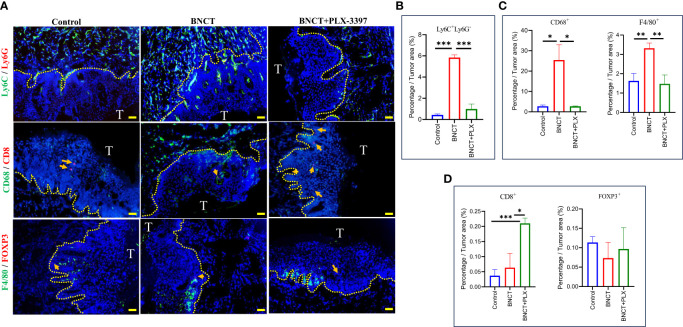
The alternations of immune profile in the tumor microenvironment following treatments. **(A)** The representative images of control, BNCT, and combined therapy-treated tumors stained with Ly6C(Green) and Ly6G(Red) at the *upper*, CD68(Green) and CD8(Red) at the *middle*, F4/80(Green) and FOXP3(Red) at the *bottom*, and nucleus (Blue) (bar=50 μm). CD8-positive (*middle*) and FOXP3-positive cells (*bottom*) were indicated by the arrows. Quantitative data from fluorescent signals showed the percentage of **(B)** Ly6C^+^LysG^-^ **(C)** CD68^+^ and F4/80^+^ **(D)** CD8^+^ and FOXP3^+^ cells within control, BNCT-, and combined therapy-treated tumors. All quantitative data represented mean ± SD. (Unpaired *t-test*,^*^ *P* < 0.05, ^**^ *P* < 0.01, ^***^ *P* < 0.001).

PLX-3397 monotherapy had no significant effect on mice survival, with a similar median survival of 40 ± 21 days compared to control mice (39 ± 14 days). However, the combination with BNCT further extended the median survival day from 66 ± 16 (BNCT monotherapy) to 90 ± 26 days (p<*, log-rank test) (**Figure 3C**). This indicates that the CSF-1R inhibitor could synergize the BNCT effect. To examine the impact of CSF-1R inhibition on the tumor microenvironment and to relate these to changes in M-MDSCs in blood, mice were sacrificed at day 28, and the immune components within the tumor microenvironment were analyzed by immunohistochemical (IHC) staining. The IHC results (**Figure 4**) showed that the administration of PLX-3397 significantly diminished BNCT-induced Ly6C^+^Ly6G^-^ M-MDSCs accumulation (**Figures 4A, B**). Since M-MDSCs could differentiate into tumor-associated macrophages (TAMs) (51), the predominant effect of PLX-3397 on TAMs was then examined. Dissecting TAMs subsets in this tumor model revealed that BNCT increased CD68^+^ and F4/80^+^ TAMs. PLX-3397 combined with BNCT reduced both TAMs subsets to levels similar to those in control tumors (**Figure 4C**). The number of CD8^+^ infiltrating lymphocyte (TIL) and Foxp3^+^ T helper cells (**Figure 4D**) were very few in control tumors and were not altered following BNCT treatment. However, we noticed that PLX-3397, combined with BNCT, caused an increase in CD8^+^ TILs. Overall, PLX-3397 could reduce BNCT-induced TAMs infiltration and increase tumor-infiltrating CD8^+^ T cells.

## Discussion

Recurrences of H&N cancer are challenging to re-treat due to tumor-surrounding tissues’ sensitivity to current treatment agents, including chemotherapeutic drugs and radiation. One potential option for treating no-option recurrences of H&N cancer is BNCT. In Taiwan, two clinical trials demonstrated the high response rate of recurrent H&N to BNCT with improved life quality (21, 52). However, recurrences are still an issue following BNCT. Combining BNCT with IMRT (intensity-modulated radiation therapy) has slight further improvement. This study explored the potential of combining immunotherapy following BNCT for H&N cancer in a murine oral cancer model.

The 4-NQO-induced oral cancer is a valuable preclinical model for studying HNSCC, but it is challenging to check the radiation effect because of the sensitivity of the oral cavity to irradiation. Using a conventional electron accelerator, 6 Gy of radiation resulted in only a 0.75-fold reduction in the surface tumor area compared to untreated tumor-bearing mice. However, this treatment also caused severe side effects that impaired the mice’s eating ability. In contrast, administering a total physical dose of 2 Gy BNCT significantly prolonged the survival duration by up to 1.7 times compared to the untreated group. Notably, this treatment exhibited the advantage of not causing side effects related to eating disorders. This indicates that BNCT is a good radiation therapy option for recurrent HNSCC, which is sensitive to RT-induced side effects, limiting the allowed RT dose. When monitoring the BNCT effect on tumor progression by blood samples, we found a transient decrease in M-MDSCs level one day after BNCT treatment, followed by a steady increase in M-MDSCs with time. The ability of BNCT to deplete intratumoral MDSCs is faster than orthotopic HNSCC mice receiving 10Gy X-ray irradiation (53). The reduction of circulating M-MDSCs on day 1 and the rise on day 7 was reflected in the change of MDSCs in BNCT-treated TME. This finding is similar to our previous study on the shift in the TME of high-dose-irradiated prostate cancer (54). Our previous study in prostate cancer has proposed that the MDSCs are a therapeutic target and an index for assessing TME. Other studies also demonstrated that blockage of MDSC infiltrating into tumors or MDSC diminishment could help to modify the tumor microenvironment (55–57). Studies on H&N cancer have also shown that the level of circulating MDSCs was associated with the duration of 4-NQO administration in the preclinical model (58) and correlated with the therapeutic response in HNSCC patients (59). MDSCs were heterogeneous cells, including at least PMN-MDSCs and M-MDSCs subsets. This study found that the major subgroup of MDSCs associated with 4-NQO-induced oral cancer progression is Ly6C^high^Ly6G^-^ M-MDSCs, not Ly6C^low^Ly6G^high^ PMN-MDSCs. Only the level of M-MDSCs, but not the PMN-MDSCs subset, is related to the tumor volume of 4-NQO-induced oral cancer. This association was also confirmed in clinical patients enrolled in our BNCT clinical trial (unpublished result). The association of M-MDCSs with tumor progression was also found in pancreatic cancer (60), non-small cell lung cancer (61), and advanced colorectal cancer patients (62), but not in prostate cancer (63), myeloma (64), and colorectal cancer patients (65), which on the other hand, have a reasonable correlation with the level of PMN-MDSCs. These results indicate that different types of tumors would recruit different subsets of MDSCs. Our previous study suggested PMN-MDSCs were the prime target for prostate cancer (59). This study proposed that M-MDSCs are a specific subset of MDSCs associated with HNSCC and could be an ideal target for treating HNSCC.

Targeting MDSCs for treating HNSCC has been proposed previously. Chen et al. used a Cox-2 inhibitor, NS-398, to reduce the recruitment of MDSCs *in vitro* and retard tumor growth in immunocompromised mice (58), but did not demonstrate its effect in 4-NQO-induced tumor growth. The impact of the Cox-2 inhibitor is mainly to reduce the inflammatory reaction resulting in broader effects on all inflammatory cells, including M-MDSCs and PMN-MDSCs. Targeting general MDSCs following radiation therapy was like our past study in high-dose-irradiated TRAMP-C1 tumors (54). However, this study found that major MDSCs in the circulation blood of 4-NQO-induced oral cancer mice affected following BNCT treatment were M-MDSCs. This finding is consistent with HNSCC patients who received medium 70Gy fractioned definitive radiation therapy with or without concurrent chemotherapy (30). This study used PLX-3397, which has a more specific effect on M-MDSCs because M-MDSCs express more CSF-1R receptors than other inflammatory cells. Our study demonstrates that the administration of PLX-3397 could effectively inhibit BNCT-induced circulating and infiltrated M-MDSCs and TAMs within the tumors, which results in the increase of CD8^+^ T-cells. This result could be attributed to the understanding that MDSCs diminish the functionality of CD8+ T cells by suppressing their proliferation and activity. Thus, by administering PLX-3397, which reduces MDSC levels, there is a potential for an increase in the population of CD8+ T cells (66–68). This could explain the synergetic effect in the group receiving the combination of BNCT with PLX-3397 treatment.

Additionally, there was a rapid reduction in M-MDSCs immediately following BNCT in this preclinical model. A similar immediate reduction in M-MDSCs was also seen in our BNCT clinical trials for recurrent H&N and glioma patients (up-published results). This indicates that high LET irradiation possesses strong tumor-shrinking capabilities and induces significant cell death in both tumor and immune cells. This effect is more apparent than conventional low LET radiation therapy (53). The resulting cell death leads to the secretion of inflammatory cytokines such as IL-34, MMP-2, and MMP-9 (69), promoting the expansion of myeloid-derived cells. Here, we discovered a subsequent increase in infiltrated Ly6C^+^Ly6C^-^ M-MDSCs up to significant tumor growth at the endpoint following BNCT. These infiltrated M-MDSCs could suppress NK cells activity (70), increase PD-1 expression to inhibit T cell proliferation (71) and activate the CSF-1/CSF-1R pathway to polarize macrophages (72), which alter the tumor microenvironment to become pro-tumor growth. This finding could explain why recurrences patients initially respond well to BNCT but subsequently experience re-recurrences near the re-irradiated sites (45, 73). These results highlight the potential of liquid biopsy, explicitly monitoring the variability of circulating M-MDSCs, as a valuable tool for assessing tumor burden in HNSCC patients. It has been demonstrated that circulating and tumor-associated M-MDSCs in HNSCC patients can suppress the function of natural killer (NK) cells (70), which are crucial immune system components. The administration of PLX-3397 significantly impacts the tumor microenvironment induced by BNCT, effectively inhibiting the population of M-MDSCs. In addition to PLX-3397, other approaches have also demonstrated efficacy in reducing the proportion of M-MDSCs within tumors. Targeting CD47 (74) and CTLA-4 (75) has been successful in this regard, as well as inhibiting the trafficking of MDSCs into tumors through CXCR1/2 blockade (70). Notably, PLX-3397 reduces the increased population of M-MDSCs following BNCT and influences the tumor microenvironment by altering macrophage polarization (76), which can support immune cell-mediated therapy. Based on the above arguments, BNCT could exhibit tumor-shrinking effects while triggering the release of pro-inflammatory cytokines like High mobility groupbox1 (HMGB1) (77).This phenomenon would foster the population of circulating M-MDSCs and draw them into the tumor milieu, promoting a pro-tumor environment. PLX-3397 would hamper the rise of circulating M-MDSCs caused by BNCT and could disrupt their infiltration into tumors. These capabilities, coupled with the FDA’s approval, underpin the potential of the BNCT+PLX-3397 combination as a strategy to inhibit the BNCT-induced rapid rebound of circulating M-MDSCs and consequently enhance the efficacy of BNCT for patients with head and neck squamous cell carcinoma.

In BNCT, the absorbed dose (Gy) can be translated into biological equivalent dose (Gy-eq) by two key factors: RBE and CBE. The Gy-eq in this study was determined using a historical equation as shown in material section. However, factors such as the boron compound used, tumor characteristics, and the tumor microenvironment can impact the CBE value, potentially resulting in overestimated or underestimated Gy absorbed doses under different circumstances (78). Previous experimental studies have demonstrated a range of CBE values, typically between 2.1 and 5.64 (79–82). In this study, we adopted a CBE value of 3.8 derived from glioblastoma, aligning with the guidelines established by the International Atomic Energy Agency (IAEA) for oral cancer (83). Notably, this value differs from the previously reported CBE value of 5.64 observed in the SCC VII-squamous carcinoma cell lines.

In summary, this study is the first to demonstrate the diversification of M-MDCSs following BNCT in the circulating system and tumors in murine HNSCC and revealed the increasing infiltrated M-MDSCs were associated with tumor burden after BNCT for HNSCC. To combine with PLX-3397, a CSF-1R inhibitor, effectively inhibits this process by blocking the signaling pathway mediated by CSF-1/CSF-1R. This combination therapy results in improved therapeutic outcomes in the context of BNCT and reveals the potential of combing M-MDCs targeting to enhance the therapeutic response of BNCT in clinical HNSCC patients.

## Data availability statement

The original contributions presented in the study are included in the article/**Supplementary Material**, further inquiries can be directed to the corresponding author/s.

## Ethics statement

The animal study was approved by Institutional Animal Care and Use Committee, National Tsing-Hua University. The study was conducted in accordance with the local legislation and institutional requirements.

## Author contributions

C-HC: Conceptualization, Data curation, Formal Analysis, Investigation, Methodology, Writing – original draft. C-JC: Data curation, Methodology, Validation, Writing – review & editing. C-FY: Formal Analysis, Funding acquisition, Writing – review & editing, Resources. H-YT: Methodology, Validation, Writing – review & editing. FC: Funding acquisition, Supervision, Validation, Writing – review & editing, Investigation, Resources, Visualization. C-SC: Conceptualization, Funding acquisition, Project administration, Supervision, Validation, Visualization, Writing – review & editing, Investigation, Resources.
