# Supplementary figures and images for: Targeting M-MDSCs enhances the therapeutic effect of BNCT in the 4-NQO-induced murine head and neck squamous cell carcinoma model
Source: Front Oncol. 2023 Oct 2;13:1263873. doi: 10.3389/fonc.2023.1263873 (PMC10598372; doi:10.3389/fonc.2023.1263873)

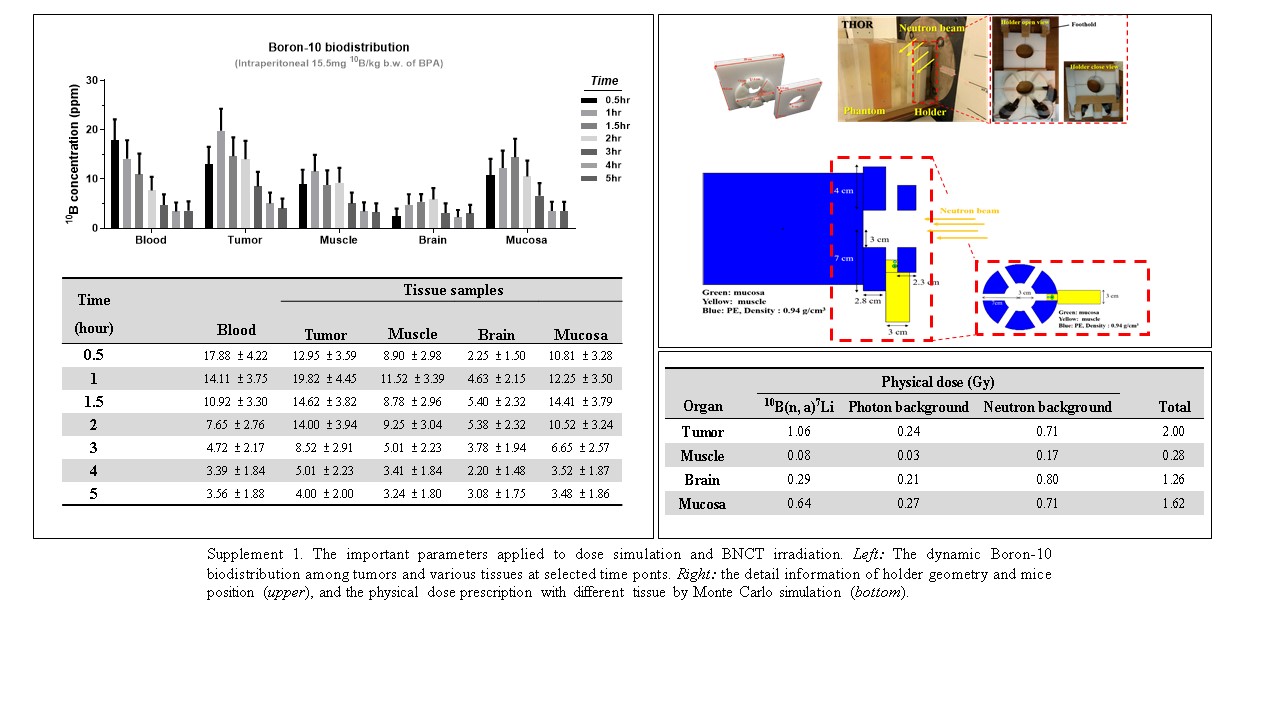

Supplement: Supplementary file 1 [file Image_1.jpeg]

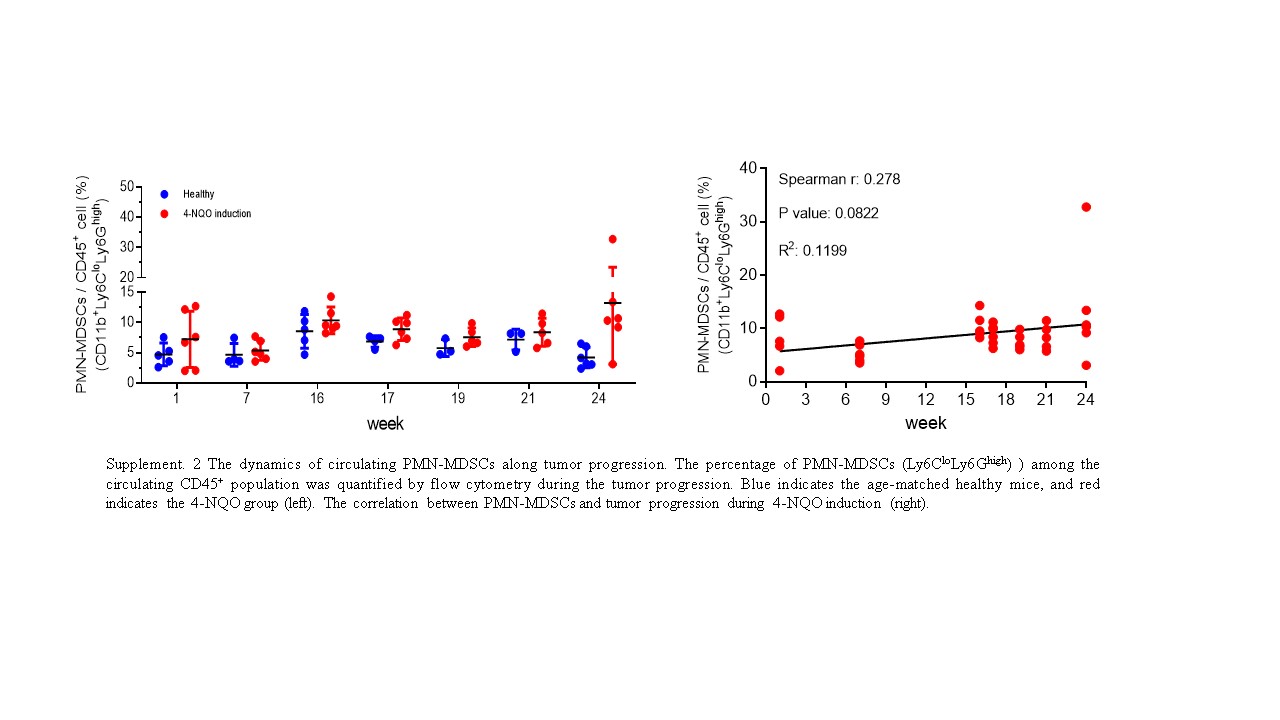

Supplement: Supplementary file 2 [file Image_2.jpeg]

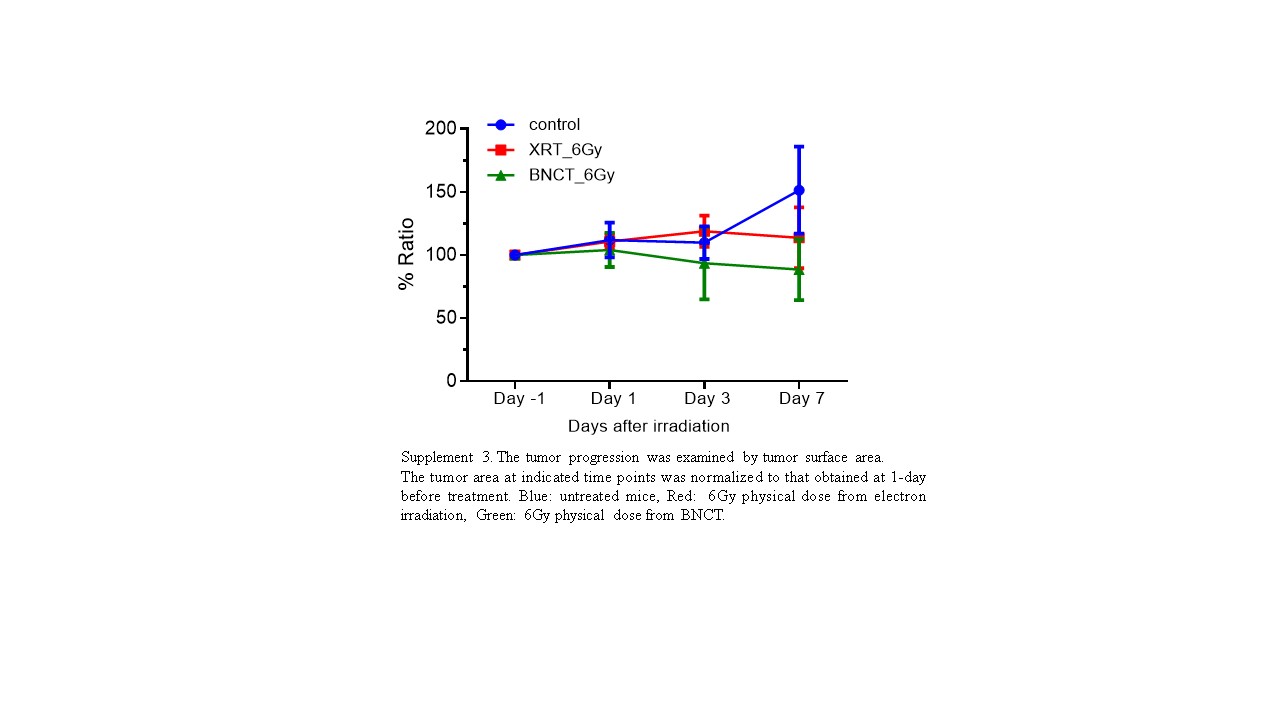

Supplement: Supplementary file 3 [file Image_3.jpeg]
